# Brain Tumor Detection Based on Deep Learning Approaches and Magnetic Resonance Imaging

**DOI:** 10.3390/cancers15164172

**Published:** 2023-08-18

**Authors:** Akmalbek Bobomirzaevich Abdusalomov, Mukhriddin Mukhiddinov, Taeg Keun Whangbo

**Affiliations:** Department of Computer Engineering, Gachon University, Seongnam-si 13120, Republic of Korea; mukhiddinov18@gachon.ac.kr

**Keywords:** brain tumor, MRI, deep learning, artificial intelligence, YOLOv7, attention mechanism, medical images, CBAM, convolution neural networks (CNN), transfer learning

## Abstract

**Simple Summary:**

In this research, we addressed the challenging task of brain tumor detection in MRI scans using a large collection of brain tumor images. We demonstrated that fine tuning a state-of-the-art YOLOv7 model through transfer learning significantly improved its performance in detecting gliomas, meningioma, and pituitary brain tumors. Our proposed deep learning model showed promising results, accurately identifying the presence and precise location of brain tumors in MRI images. The proposed approach achieved better accuracy compared to standard techniques, with a remarkable 99.5% accuracy in our analysis. However, we acknowledge that additional investigation and testing are essential to ensure the effectiveness of our method for detecting small tumors. The complexity of small tumor identification warrants ongoing research in brain tumor identification and continuous refinement of our detection systems. By pursuing this avenue, we aim to enhance the diagnostic capabilities for patients and medical practitioners in the challenging battle against brain cancers.

**Abstract:**

The rapid development of abnormal brain cells that characterizes a brain tumor is a major health risk for adults since it can cause severe impairment of organ function and even death. These tumors come in a wide variety of sizes, textures, and locations. When trying to locate cancerous tumors, magnetic resonance imaging (MRI) is a crucial tool. However, detecting brain tumors manually is a difficult and time-consuming activity that might lead to inaccuracies. In order to solve this, we provide a refined You Only Look Once version 7 (YOLOv7) model for the accurate detection of meningioma, glioma, and pituitary gland tumors within an improved detection of brain tumors system. The visual representation of the MRI scans is enhanced by the use of image enhancement methods that apply different filters to the original pictures. To further improve the training of our proposed model, we apply data augmentation techniques to the openly accessible brain tumor dataset. The curated data include a wide variety of cases, such as 2548 images of gliomas, 2658 images of pituitary, 2582 images of meningioma, and 2500 images of non-tumors. We included the Convolutional Block Attention Module (CBAM) attention mechanism into YOLOv7 to further enhance its feature extraction capabilities, allowing for better emphasis on salient regions linked with brain malignancies. To further improve the model’s sensitivity, we have added a Spatial Pyramid Pooling Fast+ (SPPF+) layer to the network’s core infrastructure. YOLOv7 now includes decoupled heads, which allow it to efficiently glean useful insights from a wide variety of data. In addition, a Bi-directional Feature Pyramid Network (BiFPN) is used to speed up multi-scale feature fusion and to better collect features associated with tumors. The outcomes verify the efficiency of our suggested method, which achieves a higher overall accuracy in tumor detection than previous state-of-the-art models. As a result, this framework has a lot of potential as a helpful decision-making tool for experts in the field of diagnosing brain tumors.

## 1. Introduction

The brain and spinal cord (together known as the Central Nervous System (CNS)) regulate many biological tasks such as organizing, analyzing, making decisions, giving orders, and integrating [1]. The human brain is incredibly complicated because of its elaborate physical make-up [2]. Stroke, infection, brain tumors, and migraines are only a few examples of CNS illnesses that present considerable difficulties in diagnosis, evaluation, and the development of effective treatments [3]. In terms of early diagnosis, brain tumors—which are caused by the abnormal proliferation of brain cells—present a significant problem for neuropathologists and radiologists. Magnetic resonance imaging (MRI) brain tumor detection is a difficult and error-prone manual process. Brain tumors are characterized by the abnormal development of nerve cells, leading to a mass. About 130 different forms of tumors can develop in the brain and CNS, ranging from benign to malignant and from extremely rare to common occurrences [4]. These malignancies can either form in the brain (primary brain tumors) or spread there from elsewhere in the body (secondary or metastatic brain tumors). Primary brain tumors refer to tumors that originate within the brain itself. These tumors are formed from the brain cells or can be encapsulated within the nerve cells surrounding the brain. Primary brain tumors can exhibit a range of characteristics, including both benign and malignant forms [5]. Secondary brain tumors, also known as metastatic brain tumors, are the most common type of malignant brain tumor. It is important to note that while benign tumors do not typically spread from one area of the body to another, secondary brain tumors are invariably cancerous and pose a serious threat to health [6].

There are a large number of patients in the United States who have been diagnosed with primary brain tumors; roughly 700,000. In addition, in the United States alone, approximately 85,000 new cases of brain tumors were detected in 2021. A patient’s age is just one of several factors that affect prognosis and survival rates when dealing with a brain tumor. Patients aged 55–64 had a 46.1% one-year survival rate, according to the research referenced in [7], whereas those aged 65–74 had a 29.3% survival rate. The authors of [8] highlight the importance of early tumor detection in increasing the likelihood of survival.

**Figure 1:**
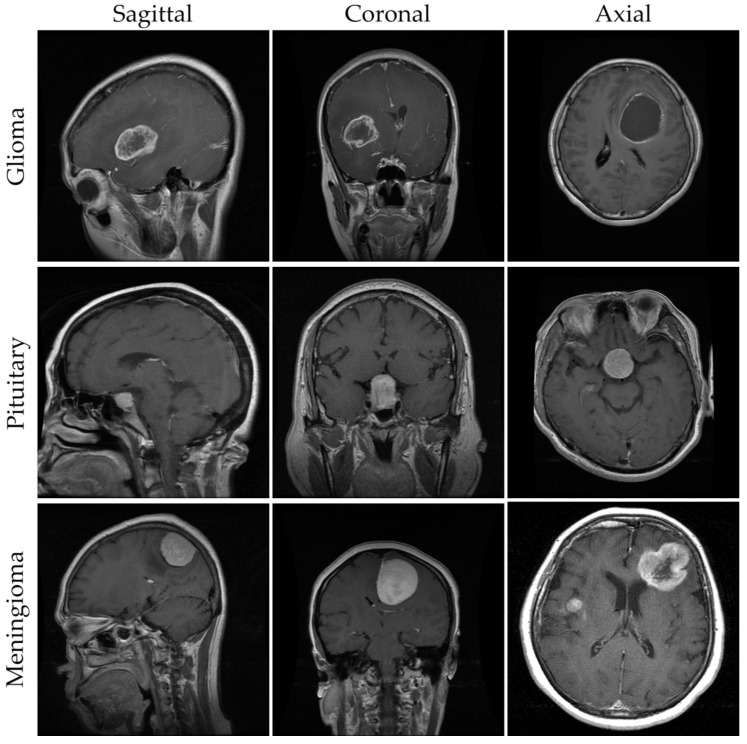
A sample of MRI images from the brain tumor dataset.

As seen in Figure 1 and described in [9], meningioma, glioma, and pituitary tumors are the most frequent primary brain tumors seen in clinical practice. Most cases of meningioma arise near the meninges tissues on the periphery of the brain or spinal cord [10]. This benign tumor develops in the membranes that rescue the brain and spinal cord. However, glioma, the brain tumor with the highest fatality rate [11], develops from the glial cells that surround and support the neurons. About a third of all cases of brain tumors are gliomas. Benign pituitary tumors develop inside the pituitary gland [12]. Prognosis and treatment options for brain tumors depend on a correct diagnosis. However, conventional biopsy techniques are painful, time-consuming, and fraught with inaccuracy in sampling [13,14]. Histopathological tumor grading (Biopsy) has its own set of problems, including intra-tumor heterogeneity and differences in the subjective assessments of different experts [15]. The diagnostic process for tumors is made more difficult and restrictive by these characteristics.

Effective treatment planning and patient outcomes depend on a quick and precise diagnosis of brain tumors. However, radiologists may spend a lot of effort on image analysis when dealing with brain tumors [16]. Today’s radiologists must rely on their own skills and subjective interpretation of pictures to make detection and decisions manually [17]. Accurate diagnosis by human visual examination alone is difficult due to the wide range of practitioners’ expertise and the inherent complexity of brain tumor images [18]. MRI scanning is commonly utilized in neurology because it allows for an in-depth examination of the skull and brain [19]. It provides axial, coronal, and sagittal imaging for a more thorough evaluation [20]. In addition to producing high-resolution pictures with great contrast, MRI also has the benefit of being a radiation-free technology. For this reason, it is the preferred noninvasive imaging technique for identifying many forms of brain malignancy [21].

Artificial intelligence (AI) plays a significant role in the detection and diagnosis of brain tumors, making it a useful complement to the notoriously difficult field of brain tumor surgery. Subsets of artificial intelligence like machine learning (ML) and deep learning (DL) have revolutionized neuropathological practices. Preprocessing of data, feature extraction, feature selection, feature reduction, and classification are only a few of the steps involved in these methods. According to ref. [22], AI has helped boost neuropathologist’s assurance in making diagnoses of brain tumors, allowing them to make better decisions for their patients.

Recent developments in deep learning have led to a wide range of useful applications [23,24] in fields as disparate as pattern classification, object detection, voice recognition, and decision making. Researchers in the healthcare industry have used a variety of ML algorithms, such as support vector machines (SVMs), k-nearest neighbor (k-NN), decision trees, Naive Bayes, and DL algorithms, such as trained convolutional neural networks (CNNs), VGGNets [25], GoogleNet [26], and ResNets [27], to aid in the diagnosis of cancer. In addition, research development is hampered by the dearth of comprehensive medical datasets due to privacy issues that prevent the exchange in patient information. In addition, because existing methods lack precision and recall, they are inefficient and take too long to classify images, which can postpone the start of treatment [28]. It can be used to diagnose neurological diseases and analyze images of brain tumor cancer [29,30].

A new automated method based on the state-of-the-art YOLOv7 model is fine tuned using our proposed module, which can replace conventional invasive brain tumor detection and enhance overall detection accuracy.

The following are the significant findings of this research:

- In order to enhance the accuracy of the brain tumor identification algorithm, a large dataset of brain tumor images was collected from open-source resources.
- To improve the readability of low-resolution MRI images, a three-stage image preparation strategy was put in place. In addition, we examined the effect of overfitting on classification accuracy and utilized a data augmentation technique to boost performance on limited datasets.
- We developed a fully automated brain tumor detection model using deep learning algorithms and YOLOv7. This model aims to reduce false detections and ultimately minimize the loss of human lives associated with brain tumors.
- After evaluating the effects of three different attention mechanisms on the model’s output, we decided to adopt the CBAM (Convolutional Block Attention Module) module. The decoupled head and CBAM attention mechanism have been verified to be successful in enhancing the performance of the brain tumor detection model.
- We integrated the SPPF+ (Spatial Pyramid Pooling Fast+) and BiFPN (Bi-directional Feature Pyramid Network) components to handle the difficulty of detecting small-size brain cancers. These modules help the model zero in on localized tumors and share the derived information across many spatial scales. Improved sensitivity to localized brain tumors is a major benefit of the BiFPN feature fusion approach that contributes to the effectiveness of the brain tumor detection model.

In the following section, we discuss previous research on brain tumors utilizing a variety of machine learning algorithms. In Section 3, we provide a detailed outline of this study, outlining both the suggested architecture and the methodology. In addition, we examine the deep learning models and performance indicators that were employed in this investigation. In Section 4, we dive into the results of our investigation of the deep learning models’ functionality. Section 5 provides a final assessment of this research and looks ahead at some of the possibilities for this area of study.

## 2. Related Works

In this section, we discuss the many ways in which machine learning and deep learning have been applied to the study of infectious brain tumors and the interpretation of medical images. Over the past two decades, medical image analysis has attracted a lot of attention and research interest because of the wide range of uses it offers in healthcare, particularly in the investigation and diagnosis of patients. In order to classify brain images and analyze brain architecture, studies suggest machine learning-based strategies [9]. Abd-Ellah et al. [31] conducted an in-depth study of the available methods for diagnosing brain MRI scans, comparing and contrasting the strengths and weaknesses of traditional machine learning and deep learning approaches. Additionally, the authors presented a new semi-automatic segmentation approach for images of brain tumors [32]. For 3D MRI segmentation, this model made use of a T1W configuration. Another CNN-based architecture was presented for breast cancer picture categorization [33]. This system’s maximum accuracy for tumor segmentation and localization was due to its architectural design, which extracted data from fitting scales. In addition, GoogLeNet, InceptionV3, DenseNet201, AlexNet, and ResNet50 were used in a CNN-based model for diagnosing brain tumors [34]. The results indicated that the proposed method was able to detect and categorize cancers in MR images with high precision. Overall, the corpus of work shows substantial advancement in segmenting and classifying brain tumors from MRI scans, as well as their 3D visualization. However, there is still a requirement for innovative methodologies to increase the efficacy of feature extraction, tumor classification, and localization.

The authors of this work [35] proposed several methods for exploiting MR images to spot brain tumors. The researchers looked into the effectiveness of using 3D CNNs, SVMs, and multi-class SVMs for segmentation improvement. In a variety of medical image processing tasks, including brain tumor detection, deep learning approaches, and CNNs in particular, showed outstanding efficacy. When compared to other machine learning classifiers, the results from using deep learning approaches to classify and segment brain tumors were superior.

An alternative study [36] presented a deep learning neural model to extract features from MR images, which were then used as input for machine learning classifiers such as Naive Bayes, Support Vector Machines, and Multilayer perceptrons. When using SVMs, the proposed method attained a classification accuracy of 96%. Kumar et al. [37] conducted a study that included the examination of several machine learning and deep learning techniques for brain tumor detection and segmentation, including support vector machines, k-nearest neighbors, multi-layer perceptrons, Naive Bayes, and random forest algorithms. Notably, the classic SVMs had the highest classification accuracy at 92.4%. For brain tumor detection in MRI, the scientists further presented a bespoke CNN architecture with five layers, which reached an amazing accuracy of 97.2%. Similarly, Khan et al. [38] developed a method for classifying and segmenting brain cancers in MRI images using VGG19 CNN architecture and K-means clustering. The suggested method first transformed the input MR modality into slices, then preprocessed the intensities using a statistical normalization strategy. The overall precision of their method was 94%.

A technique for fusing 2D and 3D MRI images was reported by the authors of [39]. They suggested utilizing DenseNet for classification and unique 3D CNN architectures for segmentation of multi-modal pictures. On the test set, the proposed method performed admirably, with an accuracy of 92% using DenseNet and 85% using the individualized 3D CNN models. Brain tumor categorization was proposed by Kang et al. [40] using machine learning classifiers and a deep CNN feature ensemble. The authors conducted their experiments using datasets of varying sizes. When compared against other machine learning and deep learning classifiers, an SVM using a radial basis function kernel performed best. Another research paper [41] developed a machine learning network-based automated brain tumor classification system for identifying high- and low-grade glioma illness images. The scientists used an extreme gradient boosting model to classify cancers of the central nervous system, including the brain, with accuracies of 90% and 95%, respectively. A new ensemble model, “Adaptive Fuzzy Deformable Fusion,” was introduced in [42]. This model improved classification and segmentation by fusing the Fuzzy C-Means Clustering technique with the deformable snake method. The experimental findings showed that the ensemble method outperformed individual models and obtained a classification accuracy of 95%. To determine how to tell benign from malignant brain tumors, Mehrotra et al. [43] investigated several pre-trained CNN algorithms based on deep learning. Various optimizers, including Adam, RMSprop, and stochastic gradient descent (SGD), were used to complete the objectives. As the research showed, when AlexNet was properly calibrated, it excelled at medical imaging tasks. To classify 253 brain tumor images (155 tumors and 98 non-tumors), Grampurohit and Shalavadi [44] built a unique CNN architecture and used VGGNet. Overfitting was mitigated by employing data augmentation and preprocessing strategies to boost sample diversity. Overall, the validation accuracy for the bespoke CNN model was 86%, whereas it was 97% for VGGNet on one dataset.

The authors of [45] reviewed several methods of image editing known as “image preprocessing”, which led to substantial enhancements in classification accuracy. Global thresholding, adaptive thresholding, the Sobel filter, the high-pass filter, median blurring, histogram equalization, dilations, and erosions were among the methods presented. In addition, 3762 pictures of brain tumors were analyzed by a pre-trained Resnet101 V2 model that relied on transfer learning to achieve a 95% rate of accuracy. Another study [46] presented a genetic algorithm (GA)–CNN hybrid for detecting glioblastoma and other brain cancers. An appropriate CNN architecture was chosen automatically with the help of the genetic algorithm in this method. The authors were able to correctly identify glioma, meningioma, and pituitary cancer in 90.9% of cases, and in 94.2% of cases overall.

Majib et al. [47] offered a novel method that combines the VGGNet architecture with a stacked classifier: VGG-SCnet. In order to facilitate faster and more effective training for automated brain tumor identification from MRI scans, the scientists fine tuned a pre-trained VGG-16 model with additional layers recommended by their approach. The most notable contours were used to pinpoint the area of interest throughout the data preparation phase. The dataset’s class imbalance was fixed using augmentation methods. The sixth layer of the VGG-16 network was used for feature extraction because it contained fewer features. Finally, tumor detection in imaging was accomplished by employing a layered classifier.

In the context of medical imaging, image preprocessing techniques are utilized to generate an accurate representation of the anatomical structure of the human body, as discussed in [48]. Specifically, in the case of MRI images, they are employed to identify and locate tumor cells within the affected human brain. In another paper [49], the authors presented a unique method that used multimodal information fusion in tandem with CNNs to spot brain cancers in 3D MRI scans. The method improved upon multimodal 3D-CNNs in order to capture the unique features of brain tumor lesions using many imaging modalities. Additionally, in [50], the researchers investigated the use of VGGNets, GoogleNets, and ResNets, among other CNN designs, in the context of brain tumor classification. According to the findings, ResNet-50 outperformed GoogleNet and VGGNets, with an accuracy rate of 96.50%, compared to 93.45% and 89.33%, respectively. In addition, ResNet-50 is 10% more accurate than VGGNet and GoogleNet while taking 10% less time to process data.

One notable study by Akkus et al. [51] focused on brain tumor segmentation using a random forest classifier. The authors utilized a combination of handcrafted features, including intensity, texture, and shape features, to train the classifier. Their approach achieved competitive performance on the BraTS 2015 (Brain Tumor Segmentation 2015) dataset, demonstrating the potential of machine learning for accurate tumor segmentation. Pereira et al. [11] proposed a deep-learning-based framework for brain tumor classification. They employed a deep CNN architecture and demonstrated superior performance in distinguishing between different tumor types compared to traditional machine learning methods.

In the procedure described in [52], a C-CNN strategy was used to zero in on the tumor-containing ROI. Both local and global features were extracted using two distinct neural network pathways in this C-CNN. The use of nested structures in the segmentation of multiple image modalities was the subject of another study [53]. A BCM-CNN strategy, tuned with a sine–cosine gray fitness algorithm, was proposed in [54]. To improve performance and decrease random errors, the strategy described in [55] used U-Net for tumor segmentation and an ensemble of models with varied hyperparameters. Complex multi-class segmentation issues were tackled by the 3D-Dense-UNet model introduced in [56]. These procedures may be time consuming and difficult to implement, and they may not be effective for some types of tumors. The approach suggested in [57] offered a new CNN and probabilistic neural network design for efficient tumor segmentation, thereby overcoming these restrictions. In addition, multiclass segmentation of three tumor regions was performed using cascaded CNNs in [58]. Most of these deep learning network models, however, were designed to perform classifications. In order to achieve correct segmentation, many existing tumor segmentation algorithms [59] require extensive quantities of training data, and they may struggle to deal with unknown tumor types in supervised learning settings.

Kamnitsas et al. [60] presented a 3D CNN architecture for brain tumor segmentation, known as “DeepMedic”. Their model utilized multi-scale inputs and incorporated contextual information through fully connected conditional random fields (CRF). The approach achieved competitive performance on the BraTS 2013 and 2014 datasets, surpassing previous methods and demonstrating the effectiveness of deep learning for accurate tumor segmentation. In another study, Havaei et al. [61] proposed a 2D and 3D deep CNN architecture for brain tumor segmentation, known as the BraTS network. Their model incorporated both local and global contextual information through multi-scale processing and achieved state-of-the-art results on the BraTS 2013 and 2015 datasets.

Furthermore, Liang et al. [62] developed a multi-modal fusion deep learning model for brain tumor grading. Their method combined features from MRI images and clinical data and achieved high accuracy in classifying tumor grades, demonstrating the potential of deep learning in aiding clinical decision-making.

## 3. Materials and Methods

### 3.1. Overall Architecture of Brain Tumor Detection

**Figure 2:**
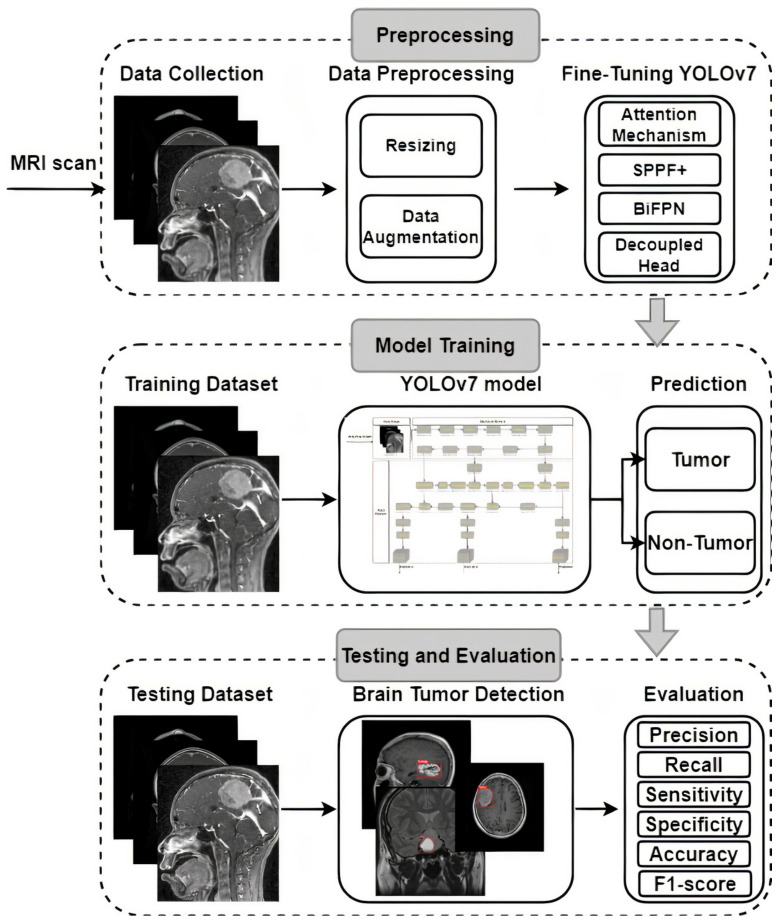
Overview of the proposed brain tumor detection based on optimized YOLOv7.

Image analysis of brain tumors is challenging, since these tumors can vary widely in size, shape, and location. Researchers have proposed several different methods for detecting anomalies in data that cannot be directly observed, each with their own set of advantages and disadvantages. The availability of a benchmark dataset capable of assessing the efficacy of state-of-the-art procedures is vital for the objective assessment of the performance of these methods. Different devices can produce brain tumor images with varying degrees of sharpness, contrast, number of slices, and pixel spacing. Here, we describe the architecture and technological details of the proposed system that make it possible to detect brain tumors in photos quickly and accurately. Brain tumor picture preprocessing, enhancements, training, and evaluation are shown in Figure 2. Several potential methods for detecting and describing brain tumors have been proposed, and these have been covered in prior research. Unfortunately, these methods have only been successfully implemented in a select few studies, with mixed outcomes at best. The suggested method’s primary focus is on providing accurate brain tumor detection in MRI scans. YOLOv7 was selected as the model to be used in this investigation because of its demonstrated efficacy in detecting brain tumors, as shown in Figure 2.

YOLOv7 is an advanced real-time object detection model that builds upon the YOLO architecture, utilizing a deep neural network to simultaneously predict bounding boxes and class probabilities for multiple objects within an input image. The YOLOv7 framework incorporates features such as the Darknet-53 backbone, PANet (Path Aggregation Network) module, and the Spatial Pyramid Pooling (SPP) layer, which collectively enhance its ability to handle scale variations and capture complex contextual information, resulting in improved detection accuracy and generalization across diverse object categories. YOLOv7 introduces progressive training with a focus on transfer learning, allowing researchers to effectively adapt the model to specific domains or datasets by leveraging pre-trained weights on larger-scale image datasets, while performing fine tuning on smaller medical or specialized datasets, making it an efficient and effective tool for various object detection tasks across domains. Pre-trained using the COCO dataset [63] to learn fundamental picture identification features and overcome a lack of training data, the YOLOv7 model was ready to be put to use.

The existing hyperparameter settings and pre-learned features from the COCO dataset made it difficult for the pre-trained YOLOv7 model to reliably identify MRI brain tumors. This effort required refining and reusing the model to make it more adept at identifying brain tumors, which were the target of interest. After the model was pre-trained and fine tuned, it was restrained with a labeled dataset of MRI brain tumors as the starting point for the new weights. As a result of these efforts, a YOLOv7-based model was developed that could recognize MRI images with meningioma, glioma, and pituitary tumors. Methods for fine tuning and transfer learning use deep learning algorithms with adjustable hyperparameters for training and improvement. For the overall loss to be minimized and the accuracy to be maximized, an optimizer that makes adjustments to the neural network’s biases and learning rate is essential. When it comes to training the model, the optimizer picked matters a lot. For this analysis, we turned to the binary cross-entropy loss function and the Adam optimizer.

### 3.2. Dataset Collection

To ensure the validity of our findings, we used an openly available MRI dataset obtained from kaggle.com [64,65]. MRI scan images are included in this collection, since they are the gold standard for diagnosing brain tumors. Glioma (2548 images), pituitary (2658 images), meningioma (2582 images), and no tumor (2500 images) were the four subsets that made up our dataset of brain tumors. Images were all scaled to 512 pixels on the horizontal and vertical dimensions. We used 8232 MRI images (or 80% of the dataset) for training in our analysis, whereas 2056 MRI images (or 20% of the dataset) were set aside for testing. Brain tumor photos from various categories are shown as examples in Figure 1. For each type of brain cancer (glioma, pituitary, and meningioma), Table 1 provides the number of pictures in various views such as axial, coronal and sagittal. It is important to keep in mind that medical photos, in contrast to natural images, are more complicated and necessitate a greater level of skill to ensure appropriate analysis and interpretation. The brain tumor dataset was labeled with oversight from a medical specialist to ensure precision and consistency. This physician’s expertise was crucial, as it established criteria for how the dataset should be labeled. However, not all brain cancers have characteristic imaging findings; therefore, depending entirely on image analysis can be risky. As a result, pathology analysis is essential for diagnosing brain cancers. Our dataset featured abnormal language descriptions annotated by a medical expert to give rich context for model training. A larger amount of training data aids in the creation of more reliable models. Data augmentation strategies can be used to increase the diversity of the training samples when the volume of available data is low. To improve a model’s generalizability, data augmentation can be used to generate new variants of the existing data. In conclusion, our model’s predictive power was enhanced by the incorporation of extensive labeled data, curated by medical experts. To further improve the prediction models’ accuracy and reliability, data augmentation techniques can be used to increase the diversity of the training samples.

The dataset was split into a training set and a testing set according to MRI view and class to ensure objective model evaluation. The efficacy of the models can then be tested on data they have never seen before, thanks to this separation into training and testing sets. This method is used to evaluate the models’ generalizability and performance in detecting brain tumor by testing them on data that has not been used in training. Testing set samples are selected blindly using stochastic collection to eliminate the possibility of bias or selection bias. This eliminates the possibility of introducing biases that might slant the evaluation results in favor of a particular model or set of assumptions.

### 3.3. Data Preprocessing and Augmentation

The brain tumor photos were subjected to a series of preprocessing stages aimed at standardizing the dataset so that it could be used in classification problems. Here is a rundown of what was undertaken in advance: The RGB photographs were converted to grayscale, creating a monochrome version of the pictures. The data were simplified, and the computing burden was lessened as a result. Images were resized such that they all had the same 640×640 resolution. This action guaranteed that all photos were the same size, guaranteeing uniformity in the subsequent processing steps. Noise in the photos was reduced and the output quality was improved by using a Gaussian blur filter. This filtering method softens the image while keeping the important details. Images were sharpened and complicated features were extracted using a high-pass filter. This filter sharpens the focus on edges and fine details, making it easier to make out critical image elements. The morphological processes of erosion and dilation were used to change the size and form of an images’ features. Erosion was used to lower the number of white areas (tumors) and highlight gaps, while dilatation was utilized to enlarge the white areas and fill gaps. Using the presence of black areas, contours were identified in the vertical, horizontal, and right-to-left directions. This process aided in locating object edges and erasing unwanted black areas from photos. This processing made the final photos suitable for feeding into the neural network models. In each photograph, Label 1 indicates a glioma tumor, Label 2 a pituitary tumor, and Label 3 a meningioma tumor. Preprocessed images and their labels were used as input material for training and assessing neural network models.

The success of the ML and DL models is highly dependent on the training data’s quality, quantity, and relevance. Yet, a lack of data is a common obstacle in the way of implementing machine learning in application. Because it is time consuming and expensive to collect useful data in many contexts, there is a dearth of it. Data augmentation methods have been created as a solution to this problem; they generate new data points from inside an existing dataset in order to artificially increase its size. To improve the model’s ability to generalize to new, unseen samples, data augmentation provides a fast and effective method for expanding the diversity of the training data. To do this, it either uses deep neural networks to generate synthetic data samples or modifies the existing data slightly. Data augmentation is becoming increasingly popular in several research areas, including signals, computer vision, speech processing, and natural language processing [66,67]. It is possible to intentionally increase the size of a dataset by employing methods like data rotation, scaling, and noise addition. Pictures can be enlarged by zooming in, flipped horizontally or vertically by certain degrees, and have their brightness altered up or down, just to name a few of the alterations that can be made. Data augmentation uses these techniques to effectively increase the training data’s dimensionality, leading to better ML and DL model performance and resilience.

Several standard computer vision techniques were used to enhance the MRI images used in this research. Model training was improved and overfitting was decreased with the help of these augmentation methods, with the aim of increasing the diversity of data samples. Various operations were performed on the original dataset, such as geometric transformations, flipping, color space conversions, random cropping, random rotations, and the introduction of noise. The trained models show improved generalizability and accuracy when applied to distributions outside the training data when this information is added to the set [68]. An open-source Python module known as Albumentations was used to perform the picture enhancements. Using the counterclockwise rotational (60 degrees and 120 degrees) and horizontal flipping methods provided by this library, a completely new set of images can be generated. Albumentations were selected with great care so that essential pixel-by-pixel information could be preserved for use in medical imaging applications [69]. In addition, the MR images were normalized using the Keras normalize function to ensure uniform pixel values for subsequent processing.

In order to identify brain tumors, we used a dataset consisting of 10,288 images. Data augmentation techniques were applied just to this fraction in order to enlarge the training set. As shown in Table 2, this brought the total number of pictures available for the task of identifying brain tumors from MRI scans to 51,448 images.

### 3.4. Network Architechture of YOLOv7

**Figure 3:**
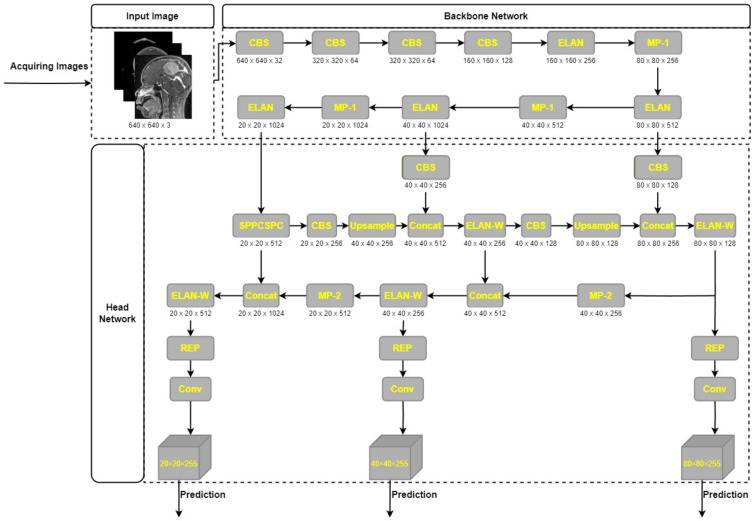
YOLOv7 network architecture [70].

Outstanding results on publicly available datasets have led to YOLOv7′s widespread recognition as a state-of-the-art object detector [70]. YOLOv7′s model architecture, depicted in Figure 3, consists of two primary parts: the Backbone network and the head network. The raw input image is preprocessed in order to prepare it for input into the Backbone network. At this point, all of the supplied pictures are resized to the standard 640 × 640 dimensions. YOLOv5′s adaptive anchor frame estimate technique, in conjunction with hybrid and mosaic data augmentation techniques, allows for this scaling up. In order to make sure the input size is suitable for the core network, this processing step is necessary. Once the input images have been properly preprocessed, it is the responsibility of the Backbone network to extract the features that are of interest. Afterwards, the head network receives these features and fuses and processes them further for object detection. Accurate object detection and localization rely heavily on the head network’s ability to fuse features into a single coherent whole.

**Figure 4:**
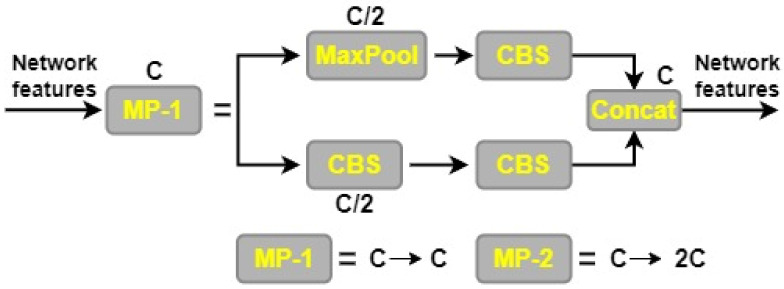
MP network architecture.

The YOLOv7 model’s backbone network is made up of the convolution, batch normalization, and SiLU (CBS) module, the MaxPool1 (MP1) module, and the extended efficient layer aggregation network (E-ELAN). This section is in charge of improving the network’s learning capacity by conducting convolution operations, normalizing the batch of data, and applying the SiLU activation function. The E-ELAN module expands on the original ELAN architecture by preserving a gradient route, which allows the network to pick up additional features via modular computation. There are two distinct parts to the MP1 module as shown in Figure 4. CBS module with 128 output channels is used in the upper branch, while a CBS algorithm with 1 × 1 stride and kernel is used in the lower branch to downsample the channel dimension, 2 × 2 stride and 3 × 3 kernel is used to downsample the width and height of the image, and a concatenation operation is used to combine the features extracted in the two branches. The CBS module with a 128-output-channel configuration and the MaxPool operation both lower the number of channels in an image while maintaining the same width and length. MaxPool and CBS operations enhance the backbone network’s capacity to discern important elements in the input image. Small local regions with maximum values are extracted by the MaxPool operation, whereas regions with minimum values are captured by the CBS operation. These processes improve the network’s overall efficiency and effectiveness by increasing its capability to extract features.

To extract and fuse features from various backbone layers, YOLOv7′s head network makes use of the Feature Pyramid Network (FPN) architecture and the extended efficient layer aggregation network (E-ELAN). Convolutional Spatial Pyramid (CSP) architecture uses the Spatial Pyramid Pooling (SPP) structure to improve feature extraction at low computational cost across many scales. The SPPCSPC module is the result of combining SPP and CSP, and it increases the network’s perceptual range as a result. The ELAN-W layer is implemented to further improve feature extraction. The MP2 block is used, which functions similarly to the MP1 block, but has two more output channels. By applying a 1 × 1 convolution to estimate the category, confidence, and anchor frame, the Rep structure is used to alter the amount of image channels in the output features. The Rep structure takes its cue from RepVGG [71] and includes a tailored residual design that simplifies actual estimations to a convolution. The network’s predictive performance is preserved, while its complexity is reduced.

### 3.5. Attention Mechanism Module

The attention mechanism is commonly used in deep learning to extract meaningful information from unstructured input. Different types of attention, such as multi-order attention, pixel attention, and channel attention, have all been used in the field of computer vision. However, methods that just consider channel attention, such as the squeeze-and-excitation (SE) technique [72], fail to account for the significance of spatial position information in visual tasks. CBAM was designed to address this shortcoming [73]. By performing global pooling on the channels, CBAM is an enhancement of the SE approach that allows for the incorporation of location data. Combining channel attention with location-based pooling (CBAM) improves the network’s ability to capture both global and local contextual information, leading to better results in visual tasks. This results in improved feature representations and enhanced discrimination between object classes, leading to better performance in complex visual recognition tasks.

**Figure 5:**
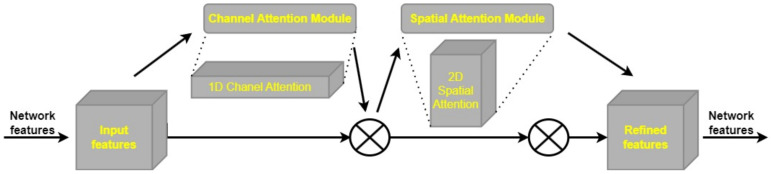
CBAM network structure [73].

The CBAM component is a fully fledged attention mechanism, successfully capturing the spatial and channel dimensions of input data. The CBAM’s Channel Attention Module (CAM) and Spatial Attention Module (SAM) are depicted in Figure 5. The CAM’s function is to bring attention to important details and regions in the channel dimension, such as the foreground and other focal points of the image. The SAM, on the other hand, is made to pinpoint where in an image crucial contextual details are located for a full picture understanding. The CBAM module, which combines the CAM and SAM, is better able to concurrently capture local and global attention, allowing the network to zero down on important spatial and channel properties. CBAM is a modular approach that can be easily integrated into various CNN architectures without significant modifications. Its flexibility allows researchers and practitioners to apply CBAM to a wide range of existing models, providing a simple and effective way to boost the performance of different network architectures across diverse computer vision tasks. By adaptively recalibrating feature maps, CBAM helps the model to focus on relevant information and suppress noise or irrelevant details, resulting in better generalization and robustness against variations in images. This adaptability is particularly valuable in scenarios with limited training data or when dealing with challenging conditions, such as different lighting conditions or occlusions. In object detection tasks, CBAM aids in accurately localizing objects of interest by enhancing spatial representations and selecting informative channels. This is especially valuable for detecting objects at different scales and in complex scenes, where objects may be occluded or appear in cluttered backgrounds. Despite its attention-based mechanism, it maintains a reasonable computational overhead, making it efficient for real-time applications. It enhances the performance of CNNs without significantly increasing the model’s computational complexity, making it suitable for resource-constrained environments.

Channel attention and spatial attention are the two components of the CBAM attention mechanism. The H × W × C input feature map is fed into a pair of 1 × 1 convolutional layers in the channel attention module, where global max pooling (GMP) and global average pooling (GAP) are used to create a pair of intermediate feature maps. After the feature maps have been generated, they are input into a two-layer multilayer perceptron (MLP) with *C*/*r* neurons (where *r* is the reduction rate) and ReLU activation in the first layer, and *C* neurons in the second layer. Both layers’ weights are uniformly dispersed across the network nodes. A sigmoid activation function is used to retrieve the channel attention feature after feature accumulation from the output. 

The spatial attention component takes the output of the channel attention feature and multiplies it, element by element, by the input feature map. The combination of channel attention and spatial attention is represented by Equation (1). To better focus on important regions of the input feature map, the model can now take into account channel-wise attention information in addition to spatial attention information obtained at this stage.

The output of the CBAM module’s channel attention module is fed into the CBAM module’s spatial attention module, which in turn generates a feature map. The output of the channel attention module is first subjected to global max pooling and global average pooling methods, which produce H × W × 1 feature maps. Dimensionality is decreased by chaining these feature maps together. Applying a sigmoid activation function to the combined feature map yields the spatial attention feature. To generate the output feature map, we multiply the input feature map by the spatial attention feature element by element. The calculation of the final feature map can be represented by Equation (2).

(1) $$M_{c}\left(F\right)=\sigma (MLP\left(AvgPool\left(F\right)\right)+MLP(MaxPool(F)))$$

(2) $$M_{s}\left(F\right)=\sigma (f^{7\times 7}\left(\left[AvgPool\left(F\right);MaxPool\left(f\right)\right]\right))$$

In order to enhance detection performance, brain tumor images can benefit greatly from the CBAM attention mechanism. The model’s capability to extract useful features and zero down on crucial parts of images is improved by the addition of CBAM. With the use of this attention mechanism, the model is better able to focus on the key locations associated with brain tumors, rather than being distracted by irrelevant information. Medical imaging of brain tumors can benefit from utilizing the CBAM attention mechanism to improve detection accuracy and reliability, leading to better performance in diagnosis and localization.

### 3.6. SPPF+

**Figure 6:**
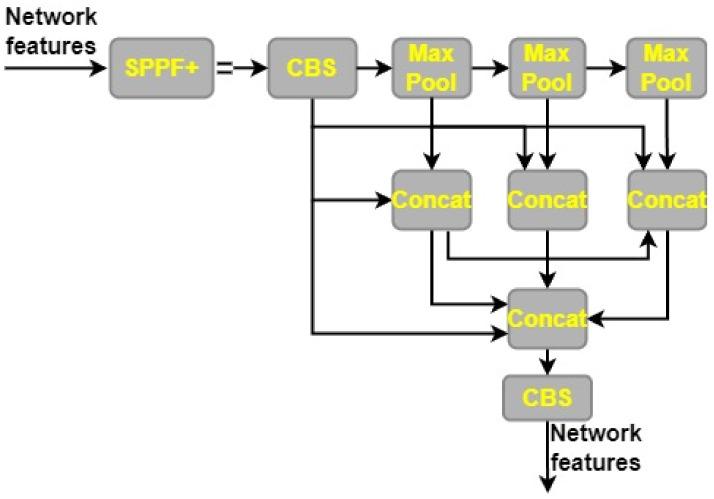
SPPF+ network structure.

We propose a new structure, SPPF+, which is an improvement on the existing Spatial Pyramid Pooling with Fusion (SPPF) architecture. SPPF+ improves upon the performance of SPPF in object detection by using the feature reuse concept from SPPF and Convolutional Spatial Pyramid Convolutional (SPPCSPC). When it comes to pooling the feature maps, the SPPF+ component uses a stride of 1 and three possible kernel sizes (13 × 13, 9 × 9, and 5 × 5). The extraction of fine-grained information from MRI is made possible because different kernel sizes correlate with different receptive fields. Improved feature interaction and pinpoint item localization are made possible by this multi-scale pooling method, which also simplifies the analysis of dense scenes. Capturing information at various sizes and combining it with global features are both parts of the process of feature learning for a picture. This is accomplished by employing strategies like maximum pooling and jump connections, which increase the feature map’s capacity for symbolic representation. For example, in max pooling, a picture is divided into several rectangular parts, and the largest value from each zone is chosen. While the pooling process helps cut down on duplicate information, it also has the potential to remove key details in some circumstances. Small targets in MRI can be found with great efficiency, because of the SPPF+ module’s ability to store global information. The SPPF+ framework is shown graphically in Figure 6.

### 3.7. BiFPN

The accuracy of object detection may suffer if there are not enough original data for training to prevent deviations throughout the learning phase. In order to overcome this difficulty, we implemented a refined version of the Bidirectional Feature Pyramid Network (BiFPN) [74] into the YOLO-V7 model’s head. Features from the feature extraction network are combined with features of relative sizes in the original BiFPN’s bottom-up pathway. The result is a two-way flow of data that makes use of connections at different scales and an extra edge. In this way, the network is able to hold onto rich semantic knowledge while still keeping track of more surface-level details. The input features to a modified version of BiFPN are given varied weights, but the same underlying structure is used several times to improve feature fusion. In this way, all of the input features may be used more effectively.

**Figure 7:**
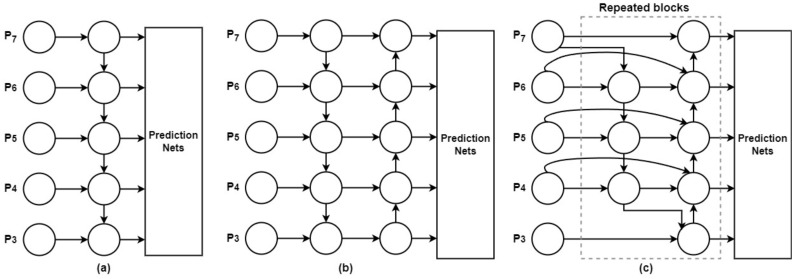
Structures of FPN (**a**), PANet (**b**), and Bi-FPN (**c**) [74].

The goal of multiscale feature fusion [75] is to combine data collected at several geographic and temporal scales. The goal is to create a mapping function *f* that efficiently merges a collection of features across scales as $P^{in}=(P_{l_{1}}^{in},P_{l_{2}}^{in},\ldots )$, indicated by $P_{l_{i}}^{in}$, into a new collection of features $P^{out}=f(P^{in})$. As shown in Figure 7a, a common method employs levels 3–7 of the input image, designated as $P^{in}=(P_{3}^{in},\ldots P_{7}^{in})$, where $P_{i}^{in}$ is the feature level retrieved from the input image and *½* reflects its size. This method is implemented using a top-down Feature Pyramid Network (FPN) [65]. For instance, if the input image is 640 × 640, a resolution of 80 × 80 pixels, $P_{3}^{in}$ would be considered feature level 3 (640/$2^{3}$ = 80), whereas a resolution of 5 × 5 pixels would be considered feature level 7. The standard FPN uses the following procedures to conduct top-down multiscale feature merging.

(3) $$P_{7}^{out}=Conv\left(P_{7}^{in}\right),$$

(4) $$P_{6}^{out}=Conv\left(P_{6}^{in}+Resize\left(P_{7}^{out}\right)\right),$$

…

(5) $$P_{3}^{out}=Conv\left(P_{3}^{in}+Resize\left(P_{4}^{out}\right)\right).$$

The *Resize* operation is commonly used in the context of multiscale feature fusion to standardize the dimensions of feature maps to ensure compatibility. Scale-independent and -dependent features can interact and blend without any hitches. To accomplish the aforementioned goals of extracting useful features and capturing spatial correlations within the feature maps, a convolutional operation (or “*Conv*” operation) is applied to each feature map. In deep learning models, feature extraction tasks typically involve a convolutional operation.

PANet is an expansion of the FPN [76] that introduces a bottom-up path assembly network to address the shortcomings of the top-down data flow in multiscale feature fusion. Figure 7b depicts an architecture that improves feature fusion. Bidirectional feature fusion is made possible by the Bi-FPN module, which draws upon level 3–7 features from the backbone network. Bi-FPN enhances the prediction of object classes and bounding boxes via the box and class networks by combining input from various scales. Bi-FPN represents an improvement over the standard FPN, since it allows for the introduction of learnable weights, making the fusion process more adaptable and effective. Bi-FPN is a more practical and efficient method than PANet, especially for real-time wildfire smoke detection, because of advancements in feature fusion. With fewer parameters and FLOPS (floating-point operations per second), Bi-FPN achieves better performance without sacrificing accuracy.

### 3.8. Decoupled Head

**Figure 8:**
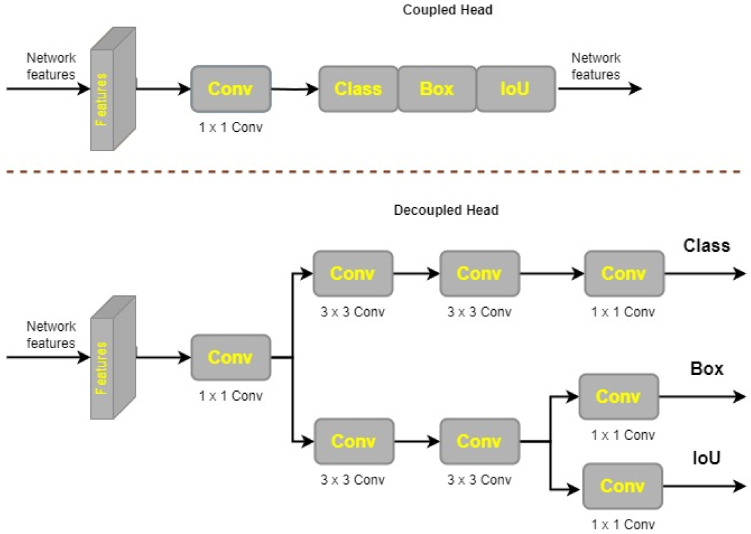
Decoupled head network structure.

As described in [77], YOLOX’s decoupled head architecture enhances detection accuracy by performing these tasks independently of one another. For prediction, regression, and classification, the decoupled head in YOLOX has more convolutional layers than the coupled head in YOLOv7. A 1 × 1 convolutional layer is used at each feature level in the decoupled head to bring the channel dimension down. The next step is split into two parallel 3 × 3 convolutional layers. There is an additional 1 × 1 convolutional layer in both forks as well. There is an IoU result in the regression tree’s structure. Figure 8 shows that there are more configuration options for the decoupled head design in YOLOX than there are for the linked head. On the other hand, it has faster convergence, which boosts accuracy performance.

### 3.9. Fine Tuning, Transfer Learning and Model Training

When it comes to deep learning tasks, poor and imprecise performance is generally the result of insufficient training data. Transfer learning, on the other hand, has emerged as a valuable technique that permits training models and producing outcomes even with insufficient data. In this article, we improve the model’s ability to detect different types of brain tumors by employing transfer learning to make use of pre-trained weights from the COCO dataset.

To further improve our model’s image recognition capabilities, we integrated characteristics learned from COCO, which are essential for tumor diagnosis. By fine tuning the model’s allocation of resources and safeguarding against memory exhaustion during training and testing, we further improve upon the pre-trained model’s performance [46]. Initial model tuning included swapping out the original eighty classes for three that more closely reflect the three types of brain cancers (glioma, pituitary, and meningioma) that were being studied. Since our desired classes have different numbers than those provided by COCO, we must make this modification.

It is also necessary to modify the model’s number of convolutional filters when changing the class size. The *ConvFilters* need to be changed from the default value of 255 to 24 to match the new class configuration, as shown in Equation (6), where *C* represents the number of classes (three in our example), 5 represents the YOLO coordinates, and 3 represents the multiple scaled bounding boxes *K*.

(6) ConvFilter=3∗(5+C)

### 3.10. Evaluation Metrics

When the training and testing phases are complete, standardized assessment criteria must be used to assess the model’s efficacy in object detection. The researchers [11,34,50] used a variety of measures for evaluation, such as precision (PR), recall (RE), sensitivity (SE), specificity (SP), accuracy (AC), F1-score, and confusion matrix (CM). These measures are calculated by applying the model to a dataset of 613 MRIs and counting the number of True Positives (TP), True Negatives (TN), False Positives (FP), and False Negatives (FN). TP represents tumor occurrences successfully identified and labeled by the model, whereas FP represents non-tumor cases incorrectly identified as tumors. Unrecognized tumors (FN) are those that were missed throughout the diagnostic process. TN stands for true negatives that were exactly as expected. Due to its measuring of the harmonic mean between FNs and FPs, the F1-score is more applicable in unbalanced datasets. Equations (7)–(11) [34,50] are used to determine each model’s accuracy, precision, sensitivity, specificity, and F1-score in order to evaluate their overall performance:

(7) $$PR=\frac{TP}{TP+FP},$$

(8) $$RE\;and\;SE=\frac{TP}{TP+FN},$$

(9) $$SP=\frac{TN}{TN+FP},$$

(10) $$AC=\frac{TP+TN}{TP+TN+FP+FN},$$

(11) $$F1-score=\frac{2(TP)}{2\left(TP\right)+FP+FN},$$

Essential criteria for assessing a model’s efficacy in the medical field include PR, RE, and F1-score. These measures shed light on how well positive predictions are made compared to total detections, how well possible positive occurrences are captured, and how well the metrics of precision and recall are balanced. Similar to traditional machine learning, these measures are essential in deep learning for evaluating a model’s performance and consistency.

## 4. Results

### 4.1. Overall Model Performance

In this section, we present the outcomes of training and verifying the suggested fine-tuned YOLOv7 model using MRI images, and we present an analysis of the overall performance. Various preprocessing and data augmentation approaches were used to increase the quality and quantity of the dataset. Various hyperparameters were used to train the suggested model for optimal results. The improved YOLOv7 model was trained on a personal computer with an 8-core 3.70 GHz CPU, Nvidia GeForce 1080Ti GPUs, and 32 GB RAM [78]. 

**Figure 9:**
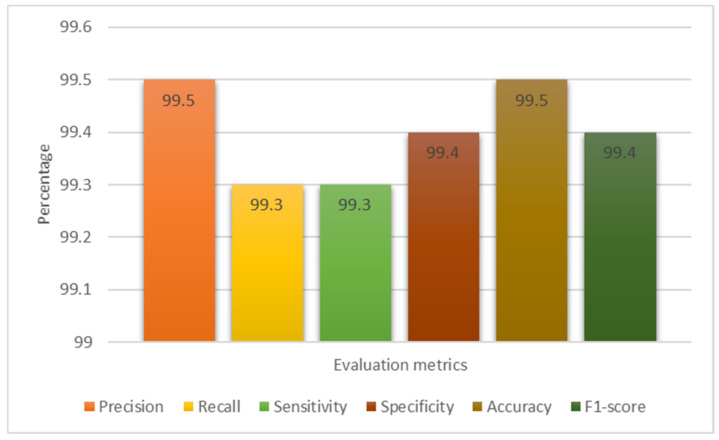
Performance evaluation of the proposed model.

**Figure 10:**
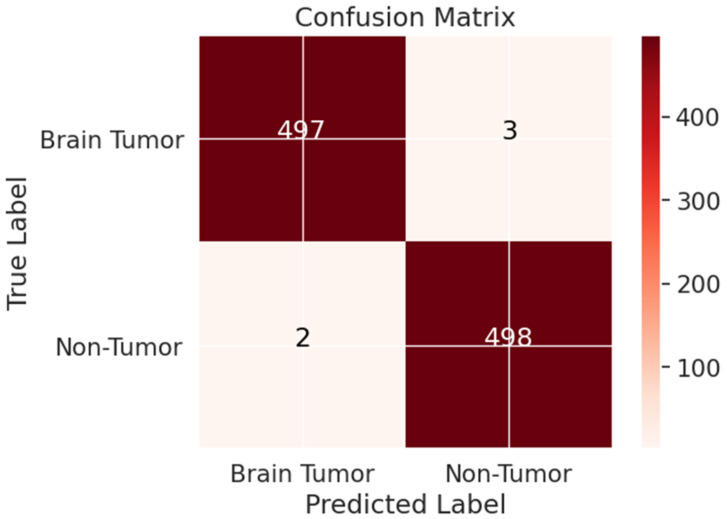
The proposed model confusion matrix.

The suggested model’s assessment metrics are displayed in Figure 9, demonstrating its efficacy in classifying data. A CM was used to estimate the model’s performance using the aforementioned assessment measures; this matrix gives insights into the number of properly identified and misclassified data samples. Figure 10 shows that the proposed model’s CM correctly detected 497 MRI images as brain tumor, while incorrectly detecting 3 MRI images. The suggested model’s assessment metrics are displayed in Figure 9, demonstrating its efficacy at detecting brain tumors.

### 4.2. Analysis of the Proposed Model in Comparison to State-of-the-Art Techniques

In this section, seven different CNN architectures were analyzed side by side: Xception, InceptionResNetV2, ResNet50, InceptionV3, VGG16, EfficientNet, and the proposed model, as shown in Table 3. While the parameters for each CNN design were the same, the number of convolutional layers and the number of fully connected (FC) layers were not. The validation accuracy and other assessment metrics for the optimized proposed network and the other pre-trained DL models used in this research are presented in Table 4. The results show that during the training phases, all models demonstrated negligible error gaps, with the exception of InceptionResNetV2, which indicated minor overfitting at the beginning. The remaining models, however, consistently minimized losses. Our suggested model, in combination with the fine-tuned YOLOv7 network, produced the best results, with 99.5% and 99.3% precision and recall, respectively. The second-best model achieved precision and recall of 97.7% and 97.9% by employing the EfficientNet architecture. The Google-created InceptionV3 model was validated at 96.4%, proving that the transfer learning strategy can be successful at spotting brain tumors. The Xception framework was able to achieve a validation accuracy of 95.6% overall. The validation accuracy of the InceptionResNetV2 method was also 96.3%. A Microsoft-created pre-trained version of ResNet50 was also employed, despite its precise performance numbers not being disclosed. These results demonstrate the efficacy and effectiveness of several CNN designs in detecting brain tumors in MR images. The suggested optimized YOLOv7 network outperformed the other assessed networks in terms of validation accuracy, making it the best-performing model. This research demonstrates the benefits of transfer learning in enhancing the precision of brain tumor diagnosis and the unique capacities of each CNN design.

The Xception method achieved the lowest accuracy (95.6%) on the validation dataset out of all the models examined in this study. When the results of each design were compared using the fine-tuned method, as shown in Table 3, it became clear that all CNNs performed well, with only subtle variances. The suggested improved YOLOv7 model achieved the most remarkable accuracy for generalizing the brain tumor pictures, beating out the other seven models. Table 4 compares the effectiveness of the model proposed in this study with that of others using machine learning (ML) and deep learning (DL) methods for detecting brain tumors. It is worth noting that direct comparisons across different studies are difficult because of differences in data preparation, training and validation procedures, and computer resources. Nonetheless, it is important to note that the model suggested in this study achieved a remarkable level of accuracy, with a 99.5% total success rate. These results demonstrate the promise of the suggested methodology for the reliable diagnosis of brain cancers. The results of different experiments should be compared with caution. Nevertheless, the obtained accuracy reflects the promise of ML and DL approaches to improving brain tumor identification and highlights the superiority of the suggested optimized YOLOv7 model.

### 4.3. Qualitative Evaluation

**Figure 11:**
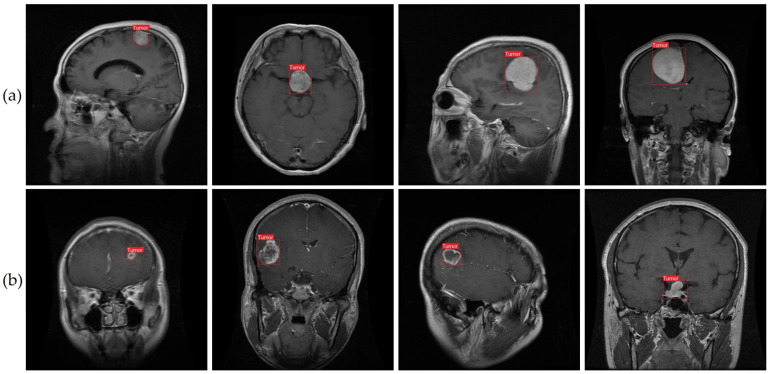
Visual representation of brain tumor detection from MRI images. (**a**) Big size of brain tumor detection, (**b**) Smaller size of brain tumor detection.

We conducted qualitative research to supplement our quantitative evaluation of our suggested technique for identifying brain tumors from MRI. To do this, we selected six MRI images from our dataset. As can be seen in Figure 11, using the improved YOLOv7 model yielded consistent and trustworthy outcomes across both classes. The situations and conditions depicted in the chosen photos covered the various range of brain tumor cases. The model’s consistent execution across a variety of settings suggests that it might be useful in monitoring and detecting brain tumors using MRI.

Previous research has struggled with the correct recognition of small brain tumors in pictures. We attempted to enlarge the dataset and enhance the accuracy of tumor detection by collecting images of brain tumors of varying sizes. In Figure 11b, examples of the smaller brain tumors that were part of our dataset can be seen. We took a cue from prior work [9] and implemented a method for accurately detecting tiny objects while still retaining fine details. The method includes merging a smaller-sized feature map with a larger-scale feature map from a higher layer. The large feature map can recognize tumor pixels of various sizes using a combination of location data from lower levels and sophisticated property data from upper layers.

Figure 11 shows that our suggested method, which employs the improved YOLOv7 model, is highly successful at detecting brain cancers under a variety of conditions. Images of both large and small tumors were used to test the consistency of our method. For effective prevention and treatment of brain cancers, early diagnosis is essential [81,82,83,84,85]. Our method successfully reduces false detections while maintaining high accuracy in locating microscopic tumor areas in pictures. Our results indicate that our proposed method shows promise as a tool for facilitating diagnosis of brain tumors and enhancing patient outcomes.

### 4.4. Ablation Study

In order to compare the efficacy of various attention mechanisms, we performed ablation tests in which we changed the CBAM (Convolutional Block Attention Module) modules and substituted the SE (Squeeze-and-Excitation) and ECA (Effective Channel Attention) mechanisms.

In order to capture the dynamic interaction between global and local data, the SE attention mechanism is built on a set of heuristics. The model is able to determine which features of an item are most important by assessing the relative weights of several channels. By focusing on the most important characteristics while ignoring the rest, the model’s performance is enhanced. As an alternative to dimensionality reduction, the ECA module provides a new method for cross-channel communication. The ECA module keeps the channel attention learning effect, unlike approaches that decrease dimensionality before collecting channel interactions. This is because it performs a local cross-channel evaluation based on the present channel and its k closest neighbors. The ECA module has been shown to be quite successful in improving model performance, despite its apparent simplicity. Our goal was to measure how these attention processes affected model performance by swapping out the CBAM modules with SE and ECA versions. The results of these studies helped us understand how various attention processes shape the model’s accuracy and information capture.

Experiments were performed on a brain tumor dataset, and the improved algorithm’s performance was measured by using the Convolutional Block Attention Module (CBAM) as the attention mechanism in the YOLOv7 model. We evaluated PR, RE, SE, SP, AC, and F1-score metrics to assess the model’s efficacy. The findings of these tests are presented in Table 5 and shed light on the improved algorithm’s efficiency.

Ablation tests were performed using the improved YOLOv7 model, which included several attention processes, including the SE and ECA modules, and the results are compared in Table 5. Taking into account the computational pressure factors, the effectiveness of these models was measured using accuracy, recall, and precision. The results show that compared to the YOLOv7 + CBAM algorithm, both the SE and ECA algorithms resulted in lower accuracy, recall, and precision scores. The computational pressure parameters of the model also increased as a result of these approaches. In contrast, the results showed that the CBAM attention mechanism, which integrates spatial and channel attention processes, performed better on average. According to the results, the CBAM attention mechanism was more effective than the SE and ECA subsystems in terms of accuracy. This suggests that the CBAM mechanism did a good job of improving the model’s capacity to zero in on significant information while ignoring noise, which ultimately led to greater accuracy in smoke detection. The results of the study emphasize the importance of selecting an appropriate attention mechanism for optimal performance of the YOLOv7 model.

Experimental ablation was used to determine how adding the SPPF+, BiFPN, and decoupled head (DP) modules affected the performance of the proposed YOLOv7 brain tumor detection model. The YOLOv7 model and its modifications using various combinations of these components were ablated eight times. The trials were carried out in a specific order: first, the original YOLOv7 model was trained with the inclusion of the SPPF+ module only, then with the BiFPN module, and finally with the DP module. In the last experiment, we trained the whole YOLOv7 model using SPPF+, BiFPN, and DP in addition to the original YOLOv7. The experiments involving ablation yielded the data shown in Table 6. The results show that the proposed modifications can improve the YOLOv7 model’s performance with the inclusion of the SPPF+, BiFPN, and DP modules. The original YOLOv7 model’s brain tumor detection accuracy was enhanced by the addition of these modules. The benefits of including these modules into the proposed model to boost performance were demonstrated by the results of the ablation tests.

Through ablation testing, the widely used object identification model YOLOv7 was found to have poor performance. These changes improve upon the original YOLOv7 model’s weaknesses and show that changes to the network’s design might boost its overall performance in object recognition.

## 5. Discussion and Future Work

This study introduced an enhanced YOLOv7 model for accurately detecting multiclass brain tumors, specifically meningioma, glioma, and pituitary tumors, as visually depicted in Figure 1 and Figure 11. Our proposed model was carefully optimized, incorporating the CBAM attention mechanism and the SPPF+ and BiFPN components. The model was trained by employing MRI images acquired through T1-weighted contrast-enhanced sequences, harnessing the enhanced anatomical insights facilitated by contrast agents.

Comparative evaluation with existing state-of-the-art models, detailed in Table 3 and Table 4, underscores the superior predictive performance of our proposed architectures. These comparisons were conducted using the same datasets and tumor types, and differed primarily in their architectural designs. The comprehensive results presented in Table 5 provide valuable insights into the impact of diverse attention mechanisms on accuracy and information capture. Additionally, the deliberate examination of SPPF+, BiFPN, and decoupled head (DP) modules, elaborated in Table 6, elucidated the individual and collective contributions of these enhancements to the performance of the optimized YOLOv7-based brain tumor detection model. This systematic ablation approach enables understanding of each component’s role, thereby guiding the refinement and efficacy optimization of this advanced brain tumor detection methodology.

Utilizing the gathered datasets [64,65], our proposed model achieved a notable prediction accuracy of 99.5%. Beyond this impressive accuracy, our model offers distinct advantages when compared to existing models. Notably, many existing methods necessitate the adoption of handcrafted feature extraction techniques [86,87,88], which might exhibit limitations when confronted with a substantial volume of images. In contrast, our proposed approach leverages learned features, obviating the need for manual feature engineering, and enhancing the adaptability and effectiveness of the model in managing extensive image datasets.

In another approach, Masood et al. adopted the Mask RCNN alongside the ResNet-50 model to detect tumor areas [89], culminating in a robust classification accuracy of 95%. Nevertheless, it is noteworthy that more advanced object detection algorithms, such as the YOLO model and Faster RCNN, surpass the performance of Mask RCNN. Gunasekara et al. employed a CNN architecture characterized by a limited number of layers and adopted the faster R-CNN approach for classifying axial MRI images into glioma and meningioma brain tumors. Their method achieved a remarkable accuracy level of 94%, while also producing bounding boxes encompassing the tumor regions [90]. The authors utilized an unsupervised active contour detection technique, specifically the Chan and Vese algorithm [91], for precise tumor border delineation. The active contour methodology harnesses energy-based forces and constraints to meticulously extract pivotal pixels, subsequently undergoing further processing and interpretation.

Nonetheless, the application of active contouring for segmentation comes with inherent limitations, including susceptibility to convergence issues within local minima during training. Additionally, there exists a potential for overlooking finer details during the energy optimization process along the entire contour path. These considerations underscore the nuances associated with employing active contour approaches in the context of medical image segmentation.

Following that, Diaz-Pernas et al. [92] employed a CNN model in their investigation, utilizing a publicly accessible dataset comprising T1-weighted contrast-enhanced MRI images. This dataset consisted of a total of 3064 slices, encompassing meningiomas (708 slices), gliomas (1426 slices), and pituitary tumors (930 slices), ultimately yielding a remarkable detection accuracy of 97%. However, it is important to highlight that the authors did not incorporate any data augmentation strategies to enhance the diversity of their dataset. In a related study, Abiwinanda et al. [93] continued along a similar path by creating a CNN model for tumor classification, using just 3064 MRI images from the Figshare dataset. Their strategy should have addressed data augmentation options, but failed to increase the dataset size. As a result, their detection accuracy was limited to 84%, trailing significantly behind results from previous research efforts. This outcome accentuates the significance of data augmentation techniques in enhancing model performance and its capacity for accurate classification.

In recent research, Ramesh et al. [94] embarked on a pioneering endeavor, in which they developed multiple deep learning models designed for post-surgical brain tumor segmentation, tailored explicitly for radiation treatment planning and longitudinal tracking. Leveraging imaging data from an anonymized repository encompassing 225 glioblastoma multiforme (GBM) patients who underwent intensity-modulated radiation therapy at Emory University over the past decade, the segmentation model was carefully trained. The standout performer, a 3D Unet architecture, demonstrated an impressive ability to accurately segment lesions in over 70% of the test cases. Notably, this model has been seamlessly integrated into the BrICS-LIT web application, catering to the needs of both clinicians and researchers. This integration greatly simplifies the application of the models within BrICS-LIT, as they only require the imaging that clinicians are actively reviewing as input. However, it is important to acknowledge that avenues for refinement still exist, particularly concerning the incorporation of additional imaging modalities. One such modality, the T1-weighted pre-contrast MRI, can substantially enhance the models’ segmentation accuracy. Addressing this potential enhancement, a plausible approach involves training generative adversarial networks using the BraTS 2021 dataset. This strategy holds the promise of further elevating the accuracy and efficacy of the segmentation models.

Our suggested model’s classification results are encouraging, but several limitations need to be appropriately addressed. As is the case with many constraints in medical imaging, one of the biggest is the need for a large and well-annotated dataset. While there is an increasing number of publicly accessible datasets, there is still a shortage of high-quality annotated information. While bounding boxes can be useful for detecting tumors, they may not be able to capture the proper borders of tumors like segmentation methods do. Due to the irregular shape of tumors, YOLO may detect additional tissue outside the defined region. Compared to classification methods, labeling datasets for use in training YOLO-based models and comparable models is a tedious and time-consuming process in and of itself. More MRI scans may be needed if YOLO proves to be sensitive to data shortages in the future. These costs are manageable in light of the benefits of the proposed solution, and YOLO can grow and change as new research is conducted to overcome its shortcomings.

Future studies can investigate if and how zero-shot learning, few-shot learning, and deep reinforcement learning (DRL) methods can be used to tackle the aforementioned issues. The problem of a lack of training data for tumor classes can be alleviated by employing zero-shot learning to construct recognition models for unseen test samples. In situations where annotated data are limited, few-shot learning approaches allow deep learning models to learn from a small number of labeled cases per class. In addition, deep reinforcement learning (DRL) may be used to lessen the need for high-quality photos and exact annotations. The absence of validation on actual clinical data should be considered a drawback of this study. While the suggested approach showed promising results on publicly accessible datasets, it still needs to be validated using data from clinical research. This shortcoming is shared by many of the other assessed models, as well as the current analysis. It would be helpful to evaluate the practical use of the suggested strategy by addressing this issue and verifying it on actual clinical data. 

We offer numerous goals and objectives for future efforts to better improve our model for classifying brain tumors. We will first put our model to the test on real-world clinical data as soon as they become available. This will allow us to directly compare our suggested model’s performance with experimental procedures and assess how well it functions in practical medical contexts. Regarding model enhancement, we will investigate more impactful deep CNN models developed for brain tumor categorization. To further our goal of precise tumor localization and delineation, we will also work on creating segmentation algorithms with decreased temporal complexity.

We readily recognize the potential benefits of expanding our dataset with a broader collection of MRI scans, which would inevitably contribute to the enhanced performance of our proposed model. In our pursuit of crafting an algorithm that demonstrates both robustness and accuracy in classifying brain tumors, the imperative lies in harnessing a larger and more diversified dataset. Prominent candidates for inclusion encompass datasets such as Brain Metastasis MRI [95] and BraTS 2021 [96]. Incorporating these datasets holds the promise of not only augmenting the model’s generalizability, but also elevating its classification precision across a broader spectrum of brain tumor variations. In addition to X-rays, CT scans, and ultrasounds, we want to eventually apply our suggested method to other medical imaging modalities. With this growth, we will be able to test how well our model performs in a wider range of medical imaging applications and inform future studies in medical image processing.

In addition, detecting small lesions is particularly challenging due to their subtle appearance and limited contrast in medical images. While deep learning models like YOLOv7 have shown promising results in object detection tasks, their effectiveness in detecting very small lesions can be uncertain, especially when the dataset lacks representative examples.

Creating synthetic images with small lesions based on the knowledge of medical experts can be valuable in enriching the dataset and ensuring that the model is exposed to cases that might not be readily available in real data. Combining the predictions of multiple detection models, each trained on different subsets of the data, can improve the overall detection performance, especially when dealing with diverse lesion sizes. Actively involving medical experts in the iterative process of annotating and selecting samples for training can help prioritize the inclusion of challenging cases with small lesions, gradually enhancing the model’s ability to detect them. Addressing the challenge of detecting very small lesions requires a comprehensive approach that involves data curation, model optimization, and expert involvement. By continually refining the detection system, researchers can work towards providing the best chances of early detection and treatment for patients with (oligo)metastases originating from non-cranial primary tumors.

## 6. Conclusions

To reduce global death rates, diagnosis of brain cancers is essential. Brain tumors can be difficult to identify because of their complex architecture, size variability, and unusual forms. In our research, we used a large collection of MRI scans of brain tumors to overcome this obstacle. We showed that a state-of-the-art YOLOv7 model could be improved by transfer learning and fine tuning in order to detect gliomas, meningioma, and pituitary brain tumors in MRI data. Our suggested CNN model demonstrates the substantial influence of deep learning models in tumor identification and demonstrates how these models have changed this field. Using a huge collection of MRI images, we found some encouraging findings in the diagnosis of brain cancers. We used a wide range of performance measures to measure the effectiveness of our deep learning models. When compared to standard techniques of categorization, the proposed technology not only detects the existence of brain tumors, but also pinpoints their precise location within the MRI images. This localization allows for fine-grained categorization without laborious human interpretation. The proposed solution, in contrast to segmentation techniques, uses a little amount of storage space and has a low computational cost, making it portable across a variety of systems. Not only did the suggested approach achieve better accuracy than prior efforts using bounding box detection techniques, it also outperformed those techniques when applied to meningioma, glioma, and pituitary brain cancers. The results were improved, and the problem was tackled with the help of picture data augmentation, even though the dataset was relatively small. Using the available data, we obtained an accuracy of 99.5% in our analysis. The proposed method for detecting brain cancers in medical images has achieved this accuracy. 

We acknowledge that additional investigation and testing are essential to validate the efficacy of our suggested method thoroughly. The domain of brain tumor identification in medical imaging remains an area of focus in research, to which end our work leverages five distinct convolutional models and transfer learning architectures. However, there is still room for further exploration and improvement in this field. The continuous advancement of brain tumor detection systems through ongoing research holds the potential to enhance diagnostic precision for patients and medical practitioners in the challenging fight against brain cancers. By refining detection systems and pushing the boundaries of knowledge in this domain, we can foster better diagnostic skills and improve patient outcomes. 

Furthermore, conducting rigorous performance evaluations on this more comprehensive dataset will provide insights into the model’s ability to differentiate between various brain lesions. While the current dataset represents an initial step towards brain tumor detection, future studies should strive to incorporate a more diverse and clinically relevant collection of brain lesions to address the complexities of real-world diagnostic challenges.

## Figures and Tables

**Table 1:** Brain tumor dataset and its specification.

Brain Tumor Dataset	Axial	Coronal	Sagittal	Total
Glioma	864	857	827	2548
Pituitary	883	885	890	2658
Meningioma	863	859	860	2582
No tumor	837	832	831	2500
Total	3447	3433	3408	10,288

**Table 2:** Data augmentation on the brain tumor dataset.

Brain Tumor Dataset	Training Images			Testing Images	Total
	Original Images	Rotated Images	Flipped Images	Original Images	
Glioma	2039	4078	6117	509	12,743
Pituitary	2066	4132	6198	592	12,988
Meningioma	2127	4254	6381	455	13,217
No tumor	2000	4000	6000	500	12,500
Total	8232	16,464	24,696	2056	51,448

**Table 3:** Evaluation performances of deep learning models and the proposed model.

Models	PR (%)	RE (%)	SE (%)	SP (%)	AC (%)	F1-Score (%)
Xception	95.7	95.9	95.9	95.4	95.6	95.8
InceptionResNetV2	96.2	96.6	96.6	96.1	96.3	96.4
ResNet50	96.6	96.8	96.8	96.2	96.5	96.7
InceptionV3	96.7	97.1	97.1	96.3	96.4	96.9
VGG16	97.4	97.7	97.7	97.3	97.6	97.5
EfficientNet	97.7	97.9	98.0	97.5	97.8	97.8
The proposed model	99.5	99.3	99.3	99.4	99.5	99.4

**Table 4:** Evaluation performances of existing state-of-the-art models and the proposed model.

Models	PR (%)	RE (%)	SE (%)	SP (%)	AC (%)	F1-Score (%)
DenseNet [39]	94.6	94.7	94.7	94.2	94.4	94.6
VGG19 [38]	95.3	95.4	95.4	95.0	94.9	95.2
CNN Ensemble [42]	95.7	95.6	95.6	95.1	95.3	95.5
Hybrid Ensemble [41]	95.6	96.0	96.0	95.3	95.2	95.7
SVM [36]	96.3	96.5	96.5	96.2	96.5	96.3
EfficientNet [79]	97.5	97.6	97.6	97.4	97.6	97.6
YOLOv4 [80]	97.6	97.8	97.8	97.5	97.5	97.8
The proposed model	99.5	99.3	99.3	99.4	99.5	99.4

**Table 5:** Comparison results of ablation experiment for attention mechanisms.

Model	Attention Mechanism			Evaluation Metrics					
	CBAM	ECA	SE	PR	RE	SE	SP	AC	F1-Score
YOLOv7	×	×	×	98.4	98.3	98.3	98.3	98.5	98.3
	√	×	×	98.9	98.8	98.8	98.7	98.9	98.7
	×	√	×	98.5	98.6	98.6	98.4	98.6	98.5
	×	×	√	98.3	98.5	98.5	98.3	98.7	98.4

**Table 6:** Comparison of the results of the ablation experiments for different modules.

Model	Modules			Evaluation Metrics					
	SPPPF+	BiFPN	DP	PR	RE	SE	SP	AC	F1-Score
YOLOv7 + CBAM	×	×	×	98.9	98.8	98.8	98.7	98.9	98.7
	√	×	×	99.1	99.0	99.0	98.9	99.1	99.0
	×	√	×	99.1	99.1	99.1	99.0	99.2	99.1
	×	×	√	99.1	99.1	99.1	99.0	99.2	99.0
	√	√	×	99.4	99.2	99.2	99.2	99.3	99.2
	×	√	√	99.4	99.2	99.2	99.3	99.3	99.3
	√	×	√	99.3	99.2	99.2	99.2	99.3	99.2
	√	√	√	99.5	99.3	99.3	99.4	99.5	99.4

## Data Availability

The data used in this study is openly available and can be accessed from the following source: [64,65].
